# Supplementary material for: In Vitro and In Silico Study of the α-Glucosidase and Lipase Inhibitory Activities of Chemical Constituents from Piper cumanense (Piperaceae) and Synthetic Analogs
Source: Plants (Basel). 2022 Aug 24;11(17):2188. doi: 10.3390/plants11172188 (PMC9460781; doi:10.3390/plants11172188)
Supplement: Supplementary file 1 [file plants-11-02188-s001.zip › plants-1867094-supplementary.pdf]

plants

Article

In Vitro and In Silico Study of the α-Glucosidase and Lipase Inhibitory Activities of Chemical Constituents from *Piper cumanense* (Piperaceae) and Synthetic Analogs

Juliet A. Prieto-Rodríguez [1,*], Kevin P. Lévuok-Mena [1], Juan C. Cardozo-Muñoz [2], Jorge E. Parra-Amin [3], Fabián Lopez-Vallejo [4], Luis E. Cuca-Suárez [2] and Oscar J. Patiño-Ladino [2]

1 Departamento de Química, Facultad de Ciencias, Pontificia Universidad Javeriana, Bogotá 110231, Colombia
2 Departamento de Química, Facultad de Ciencias, Universidad Nacional de Colombia, Sede Bogotá, Bogotá 111321, Colombia
3 Facultad de Ciencias, Universidad de Ciencias Aplicadas y Ambientales, Bogotá 111166, Colombia
4 Departamento de Física y Química, Facultad de Ciencias Exactas y Naturales, Universidad Nacional de Colombia-Sede Manizales, Kilómetro 9 vía al aeropuerto, La Nubia, Manizales 170003, Colombia
* Correspondence: juliet.prieto@javeriana.edu.co; Tel.: +57-6013208320 (ext. 4124)

Supplementary Materials

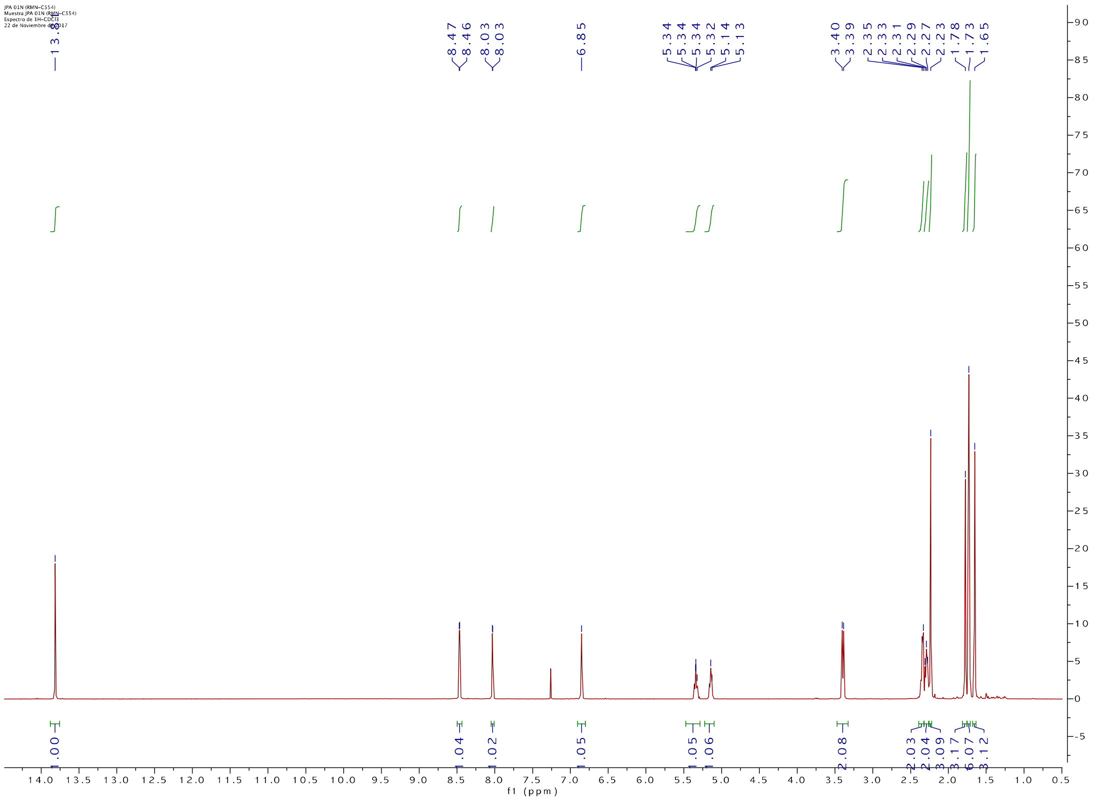

Figure S1. 1H -NMR spectra of (2′*E*) Cumenic acid (**1**).

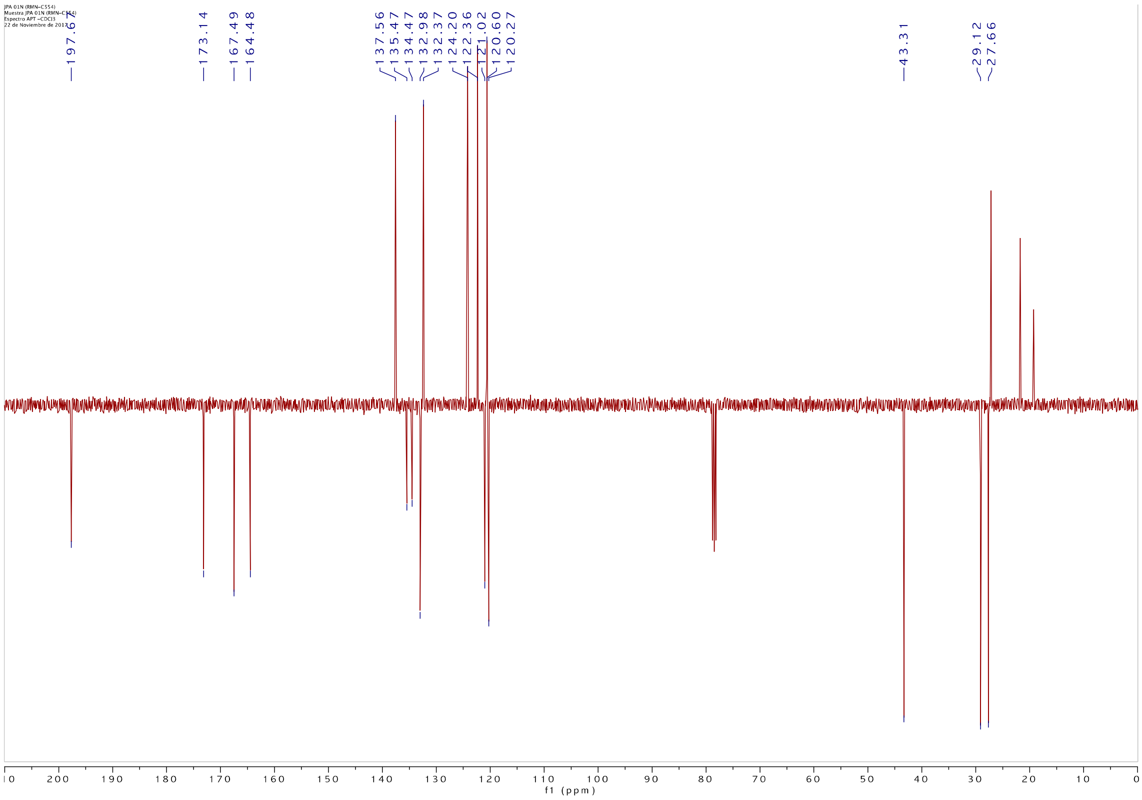

Figure S2. APT spectra of (2′*E*) Cumenic acid (**1**).

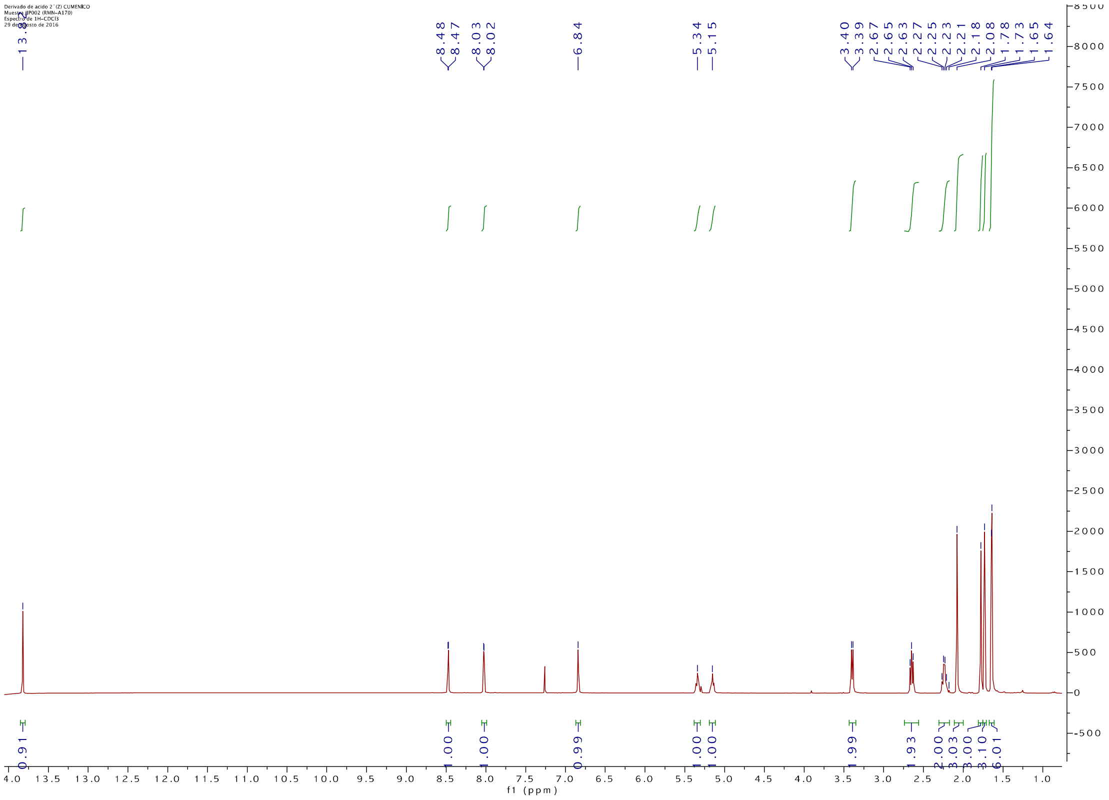

Figure S3. 1H -NMR spectra of (2′*Z*) Cumenic acid (**2**).

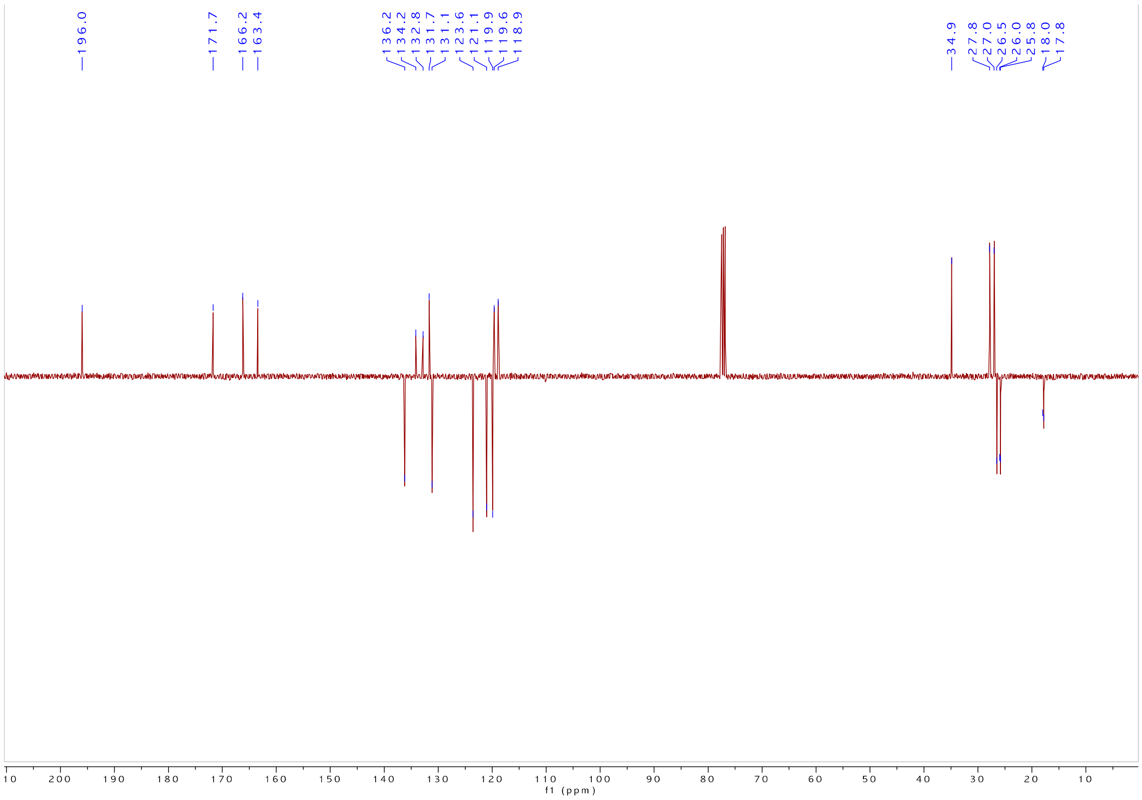

Figure S4. APT spectra of (2′Z) Cumenic acid (**2**).

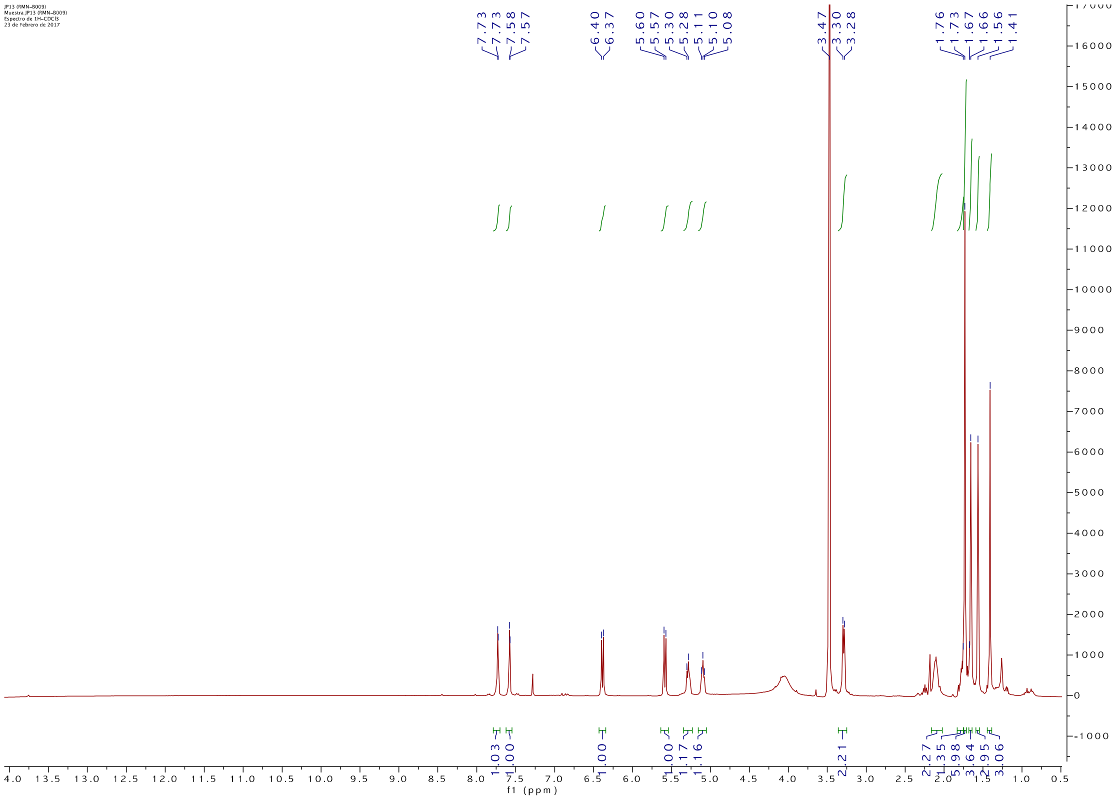

Figure S5. 1H -NMR spectra of Gaudichaudianic acid (**3**).

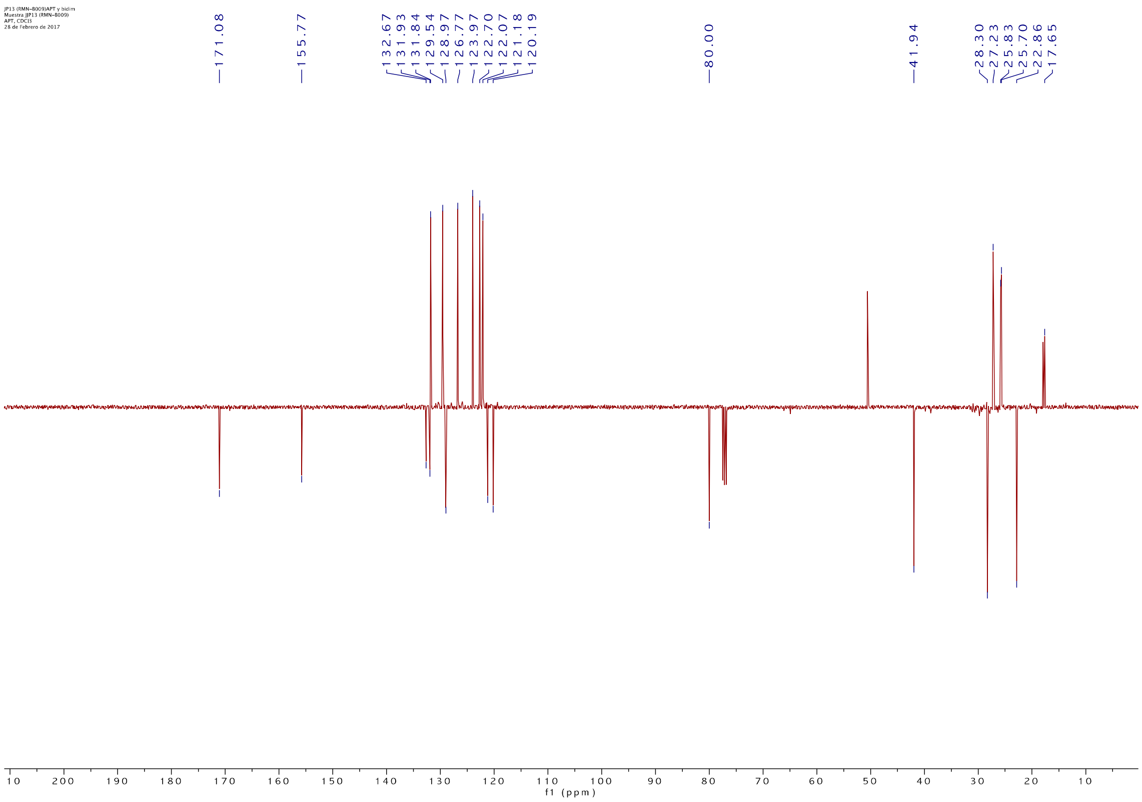

Figure S6. APT spectra of Gaudichaudianic acid (**3**).

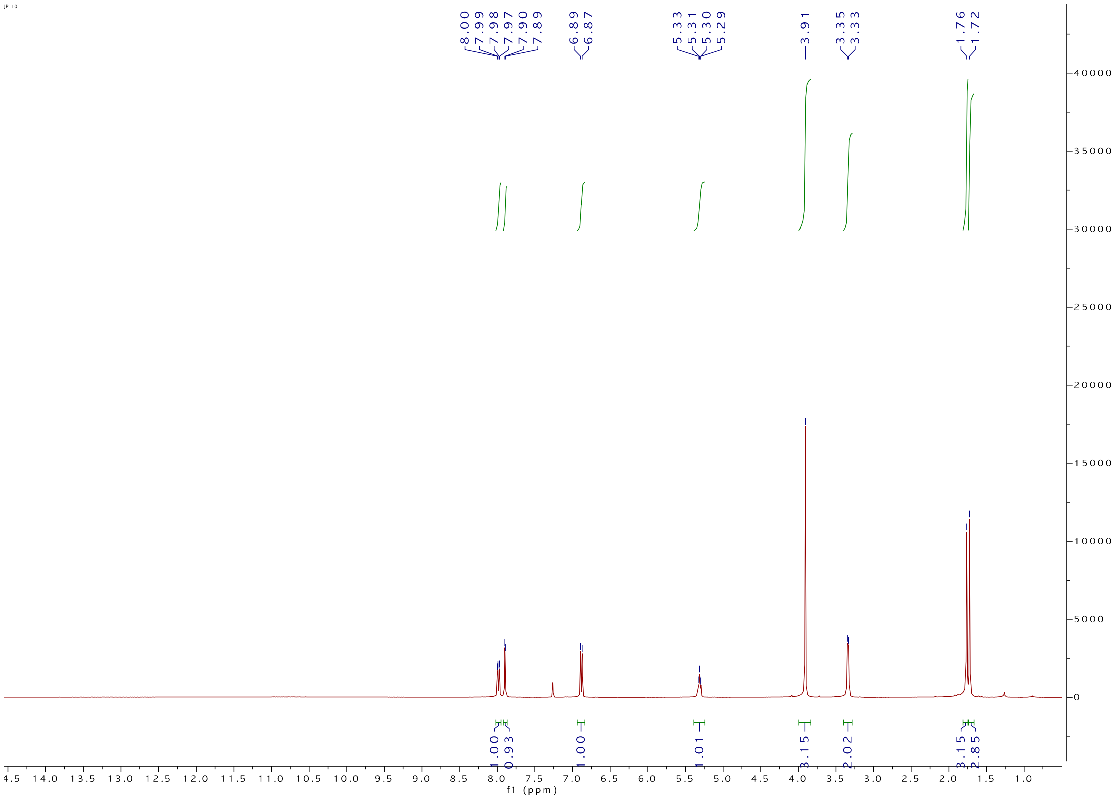

Figure S7. 1H -NMR spectra of 4-Methoxy-3-(3′-methyl-2-butenyl)benzoic acid (**4**).

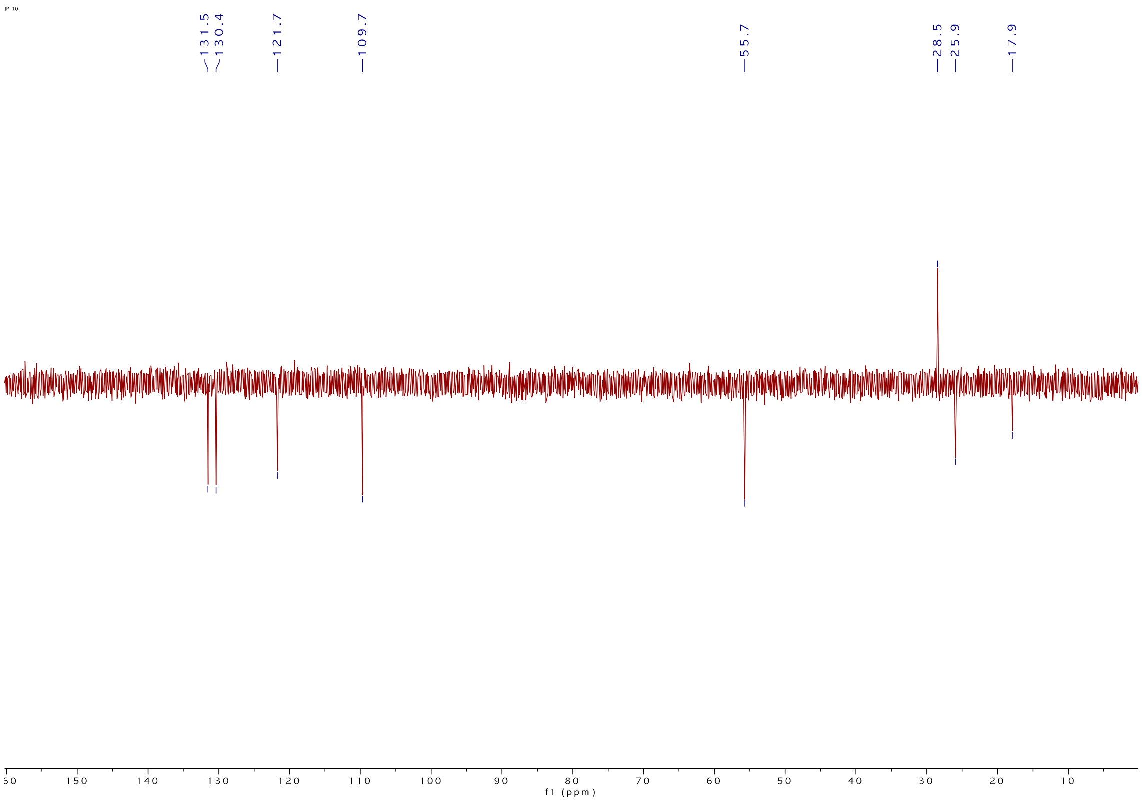

Figure S8. APT spectra of 4-Methoxy-3-(3′-methyl-2-butenyl)benzoic acid (**4**).

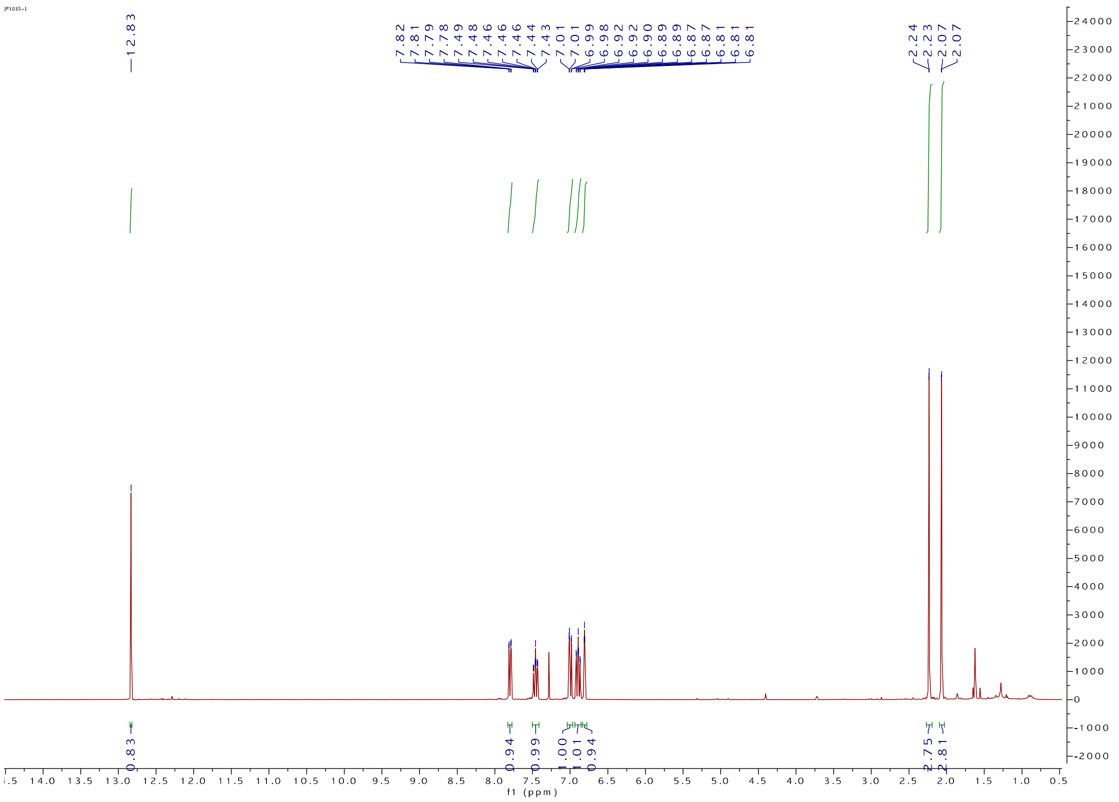

Figure S9. 1H -NMR spectra of 1-(2-Hydroxy-phenyl)-3-methyl-but-2-en-1-one (**5**).

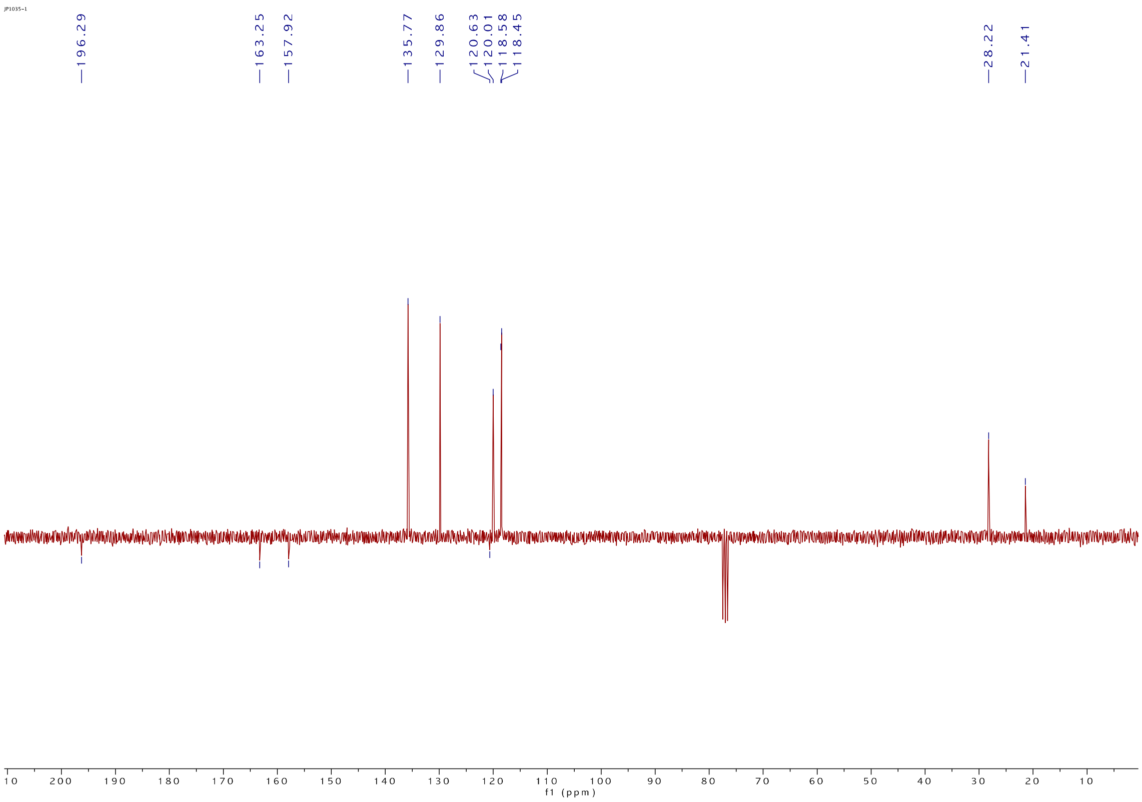

Figure S10. APT spectra of 1-(2-Hydroxy-phenyl)-3-methyl-but-2-en-1-one (**5**).

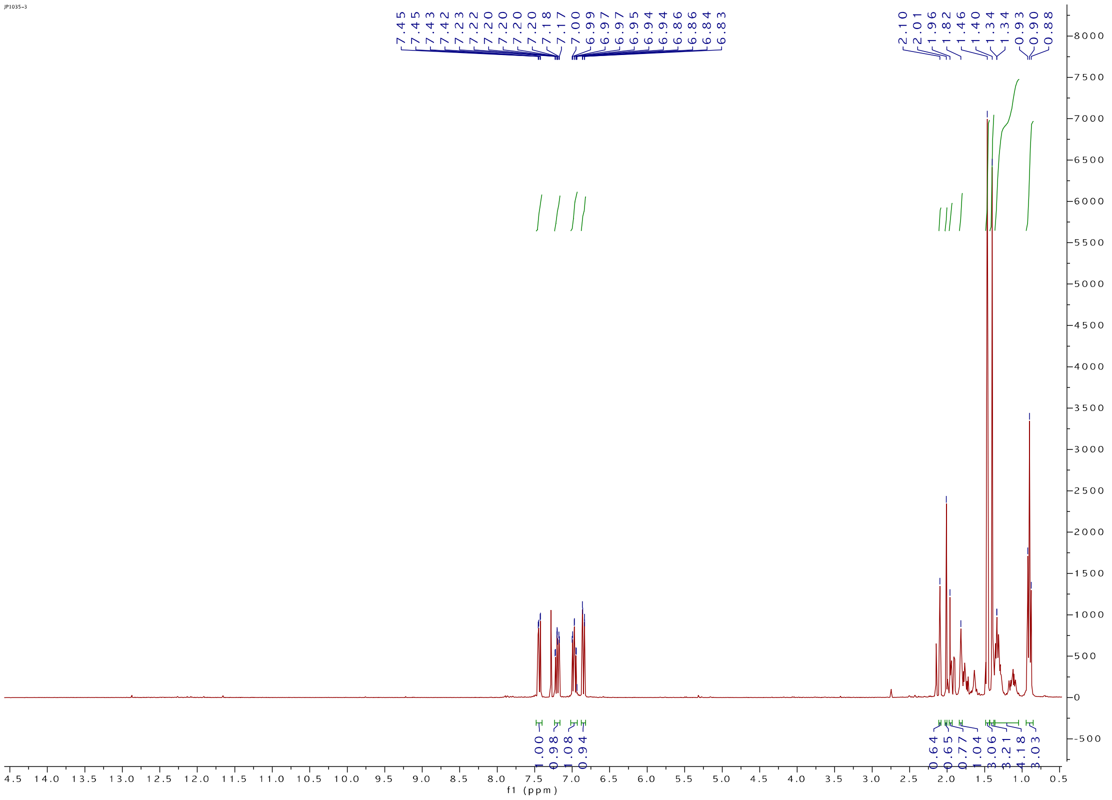

Figure S11. 1H -NMR spectra of 4-Butyl-2,2-dimethyl-chroman-4-ol (**6**).

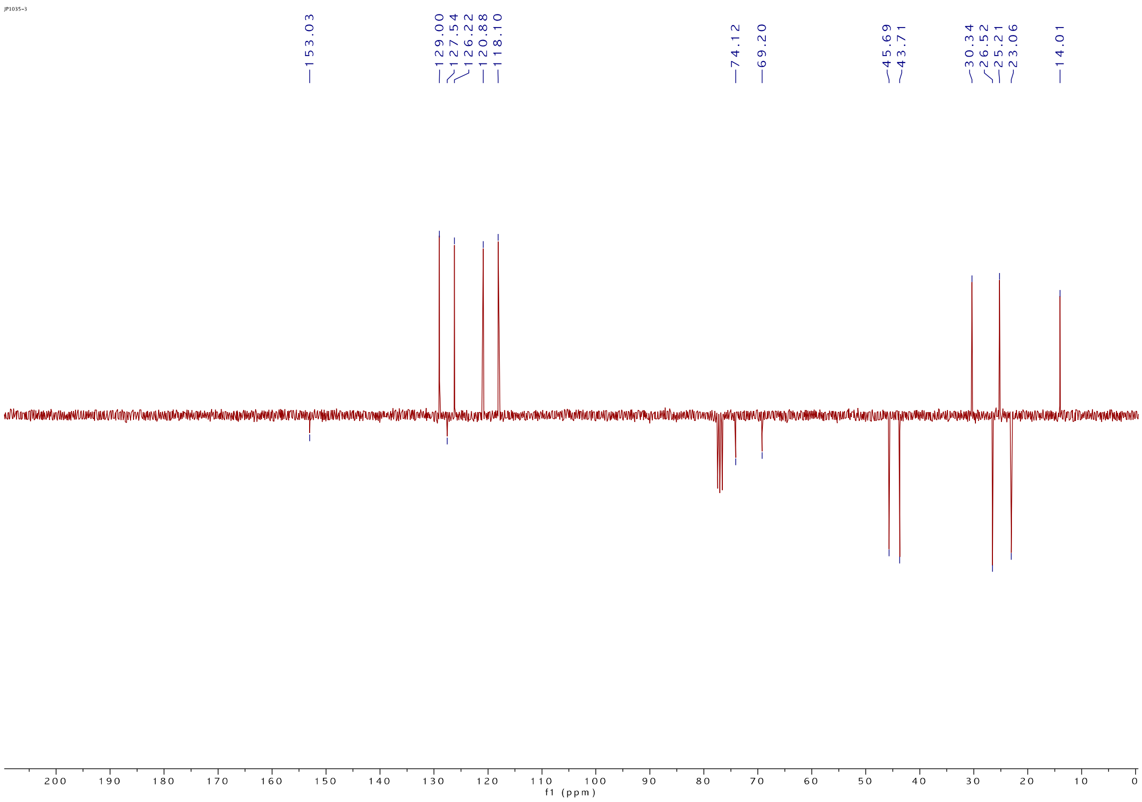

Figure S12. APT spectra of 4-Butyl-2,2-dimethyl-chroman-4-ol (**6**).

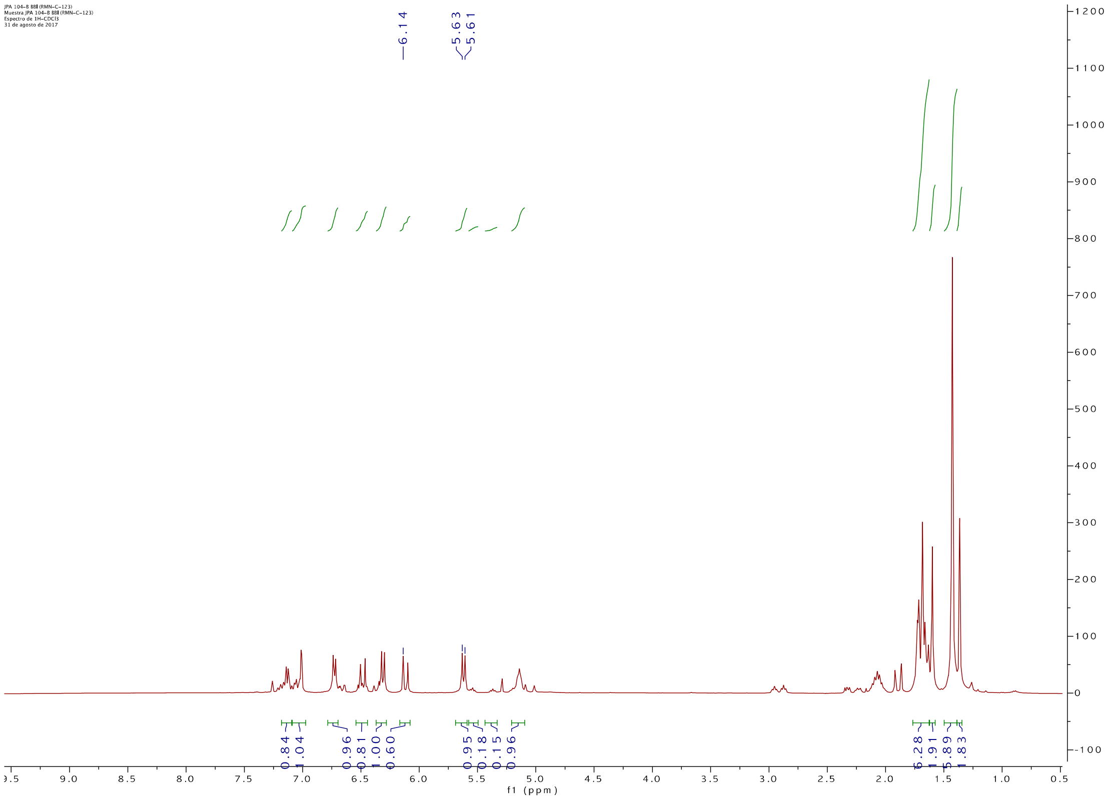

Figure S13. 1H -NMR spectra of 1-(2,2- dimethyl-chroman-6-yl)-3,7-dimethyl-octa-2,6-dien-1-ol (**7**)

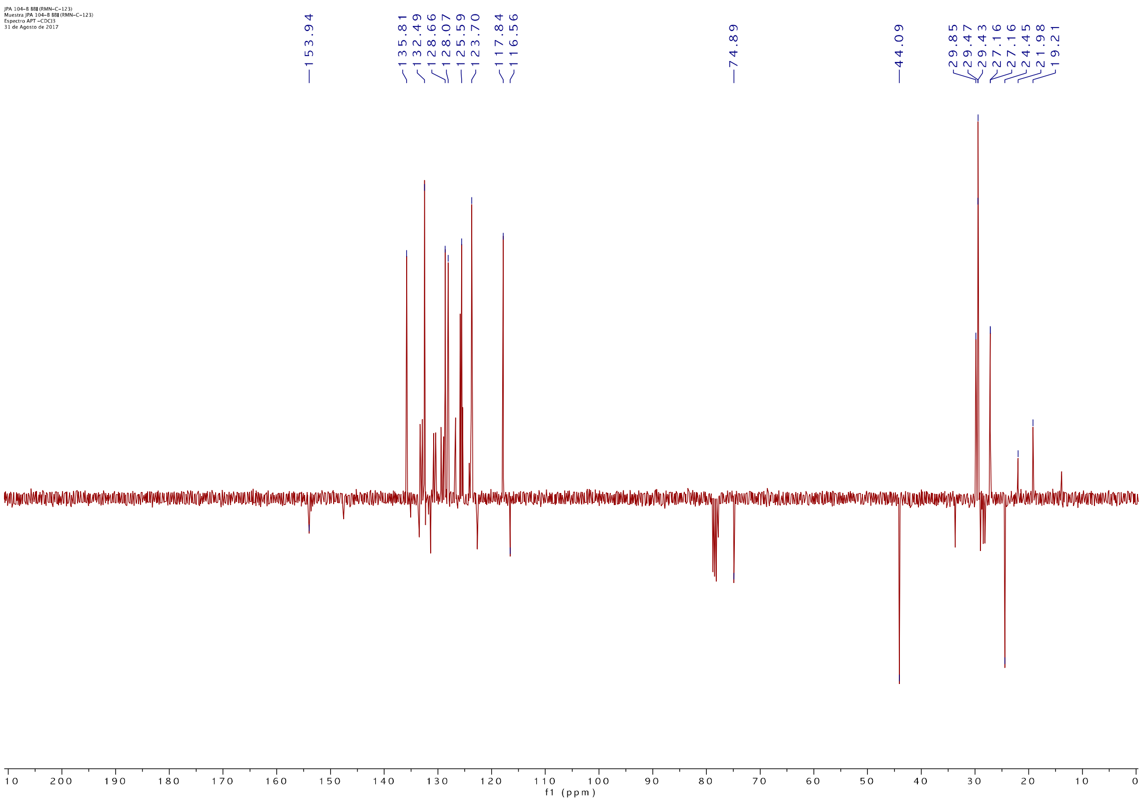

Figure S14. APT spectra of 1-(2,2- dimethyl-chroman-6-yl)-3,7-dimethyl-octa-2,6-dien-1-ol (**7**).

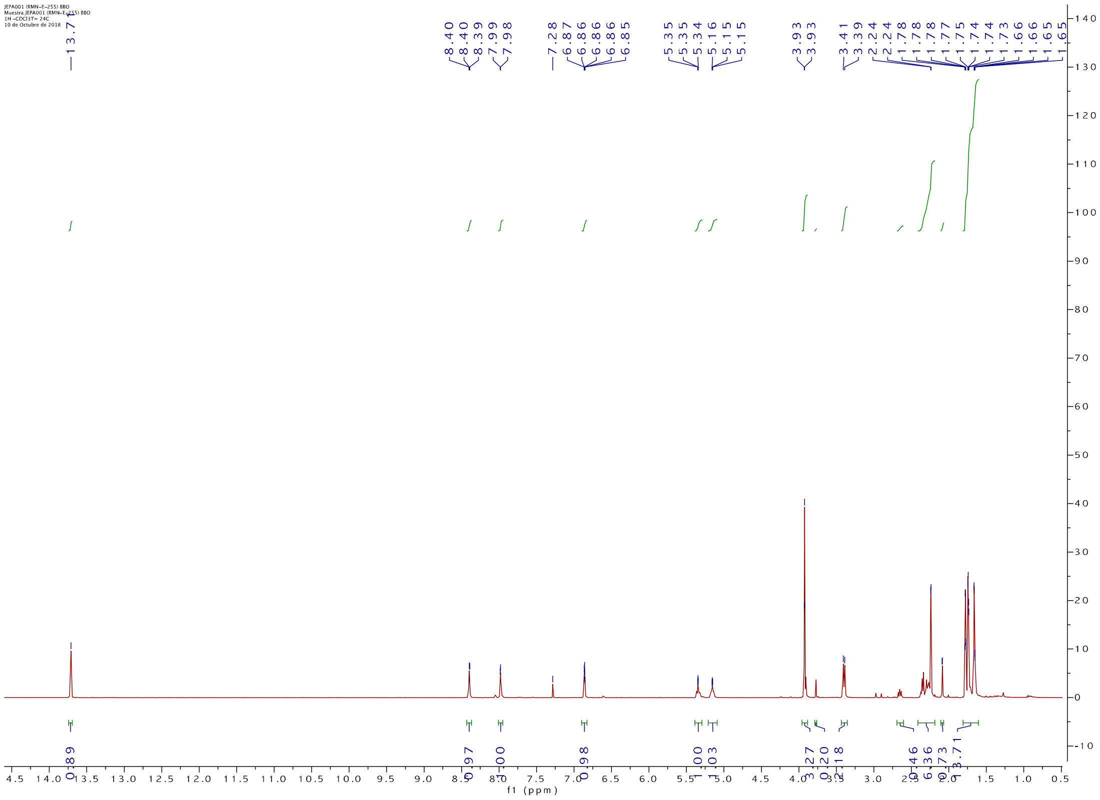

Figure S15. 1H -NMR spectra of methyl (E)-3-(3,7-dimethylocta-2,6-dienoyl)-4-hydroxy-5-(3-methylbut-2-en-1-yl)benzoate (**8**).

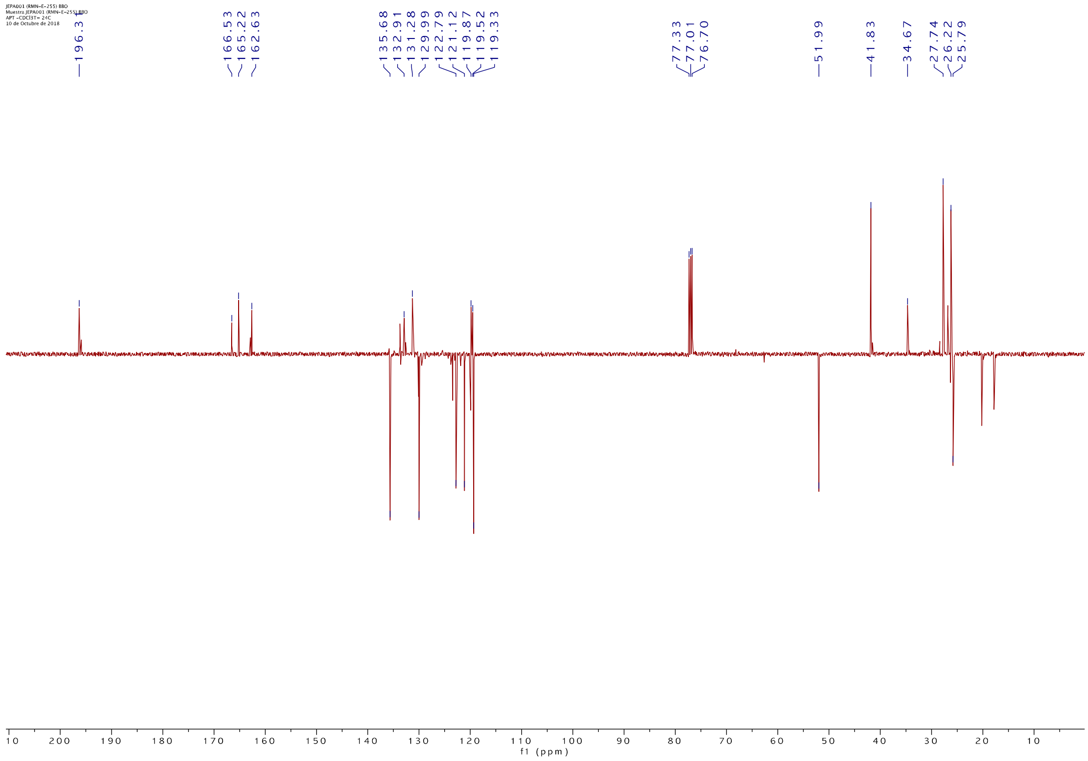

Figure S16. APT spectra of methyl (E)-3-(3,7-dimethylocta-2,6-dienoyl)-4-hydroxy-5-(3-methylbut-2-en-1-yl)benzoate (**8**).

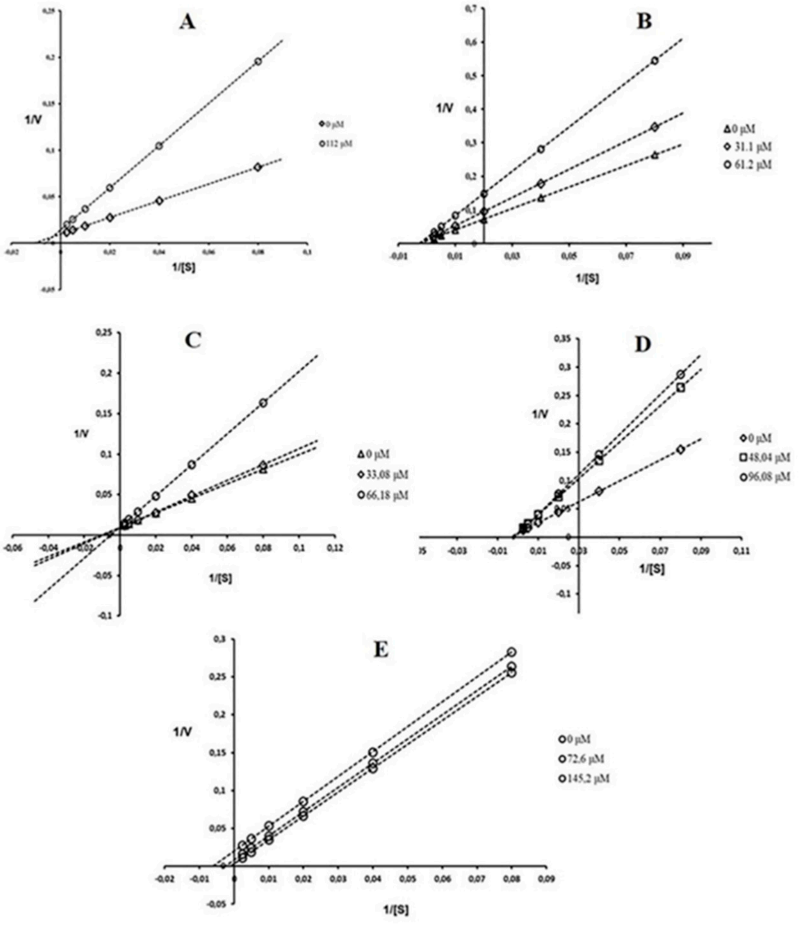

Figure S17. Mechanisms of inhibition of the catalytic activity of the PL.

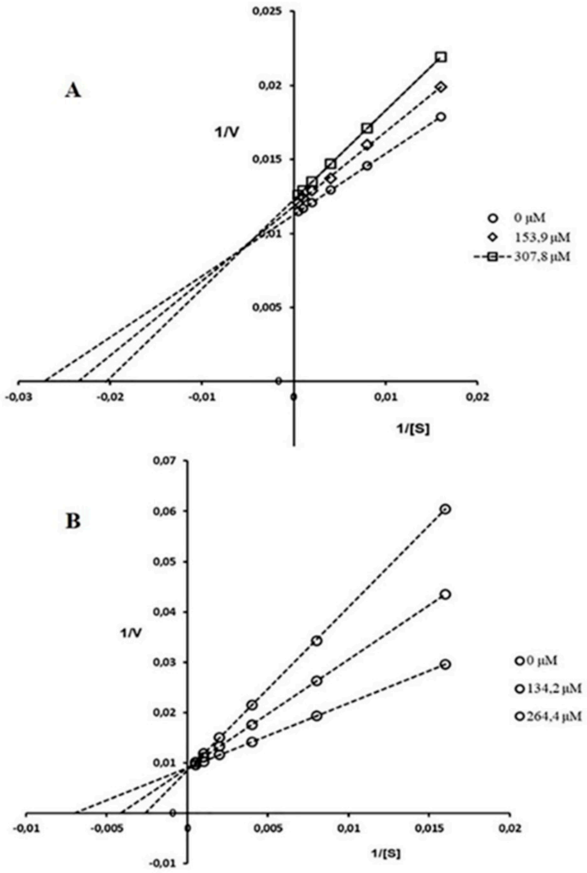

Figure S18. Mechanisms of inhibition of the catalytic activity of the AG.

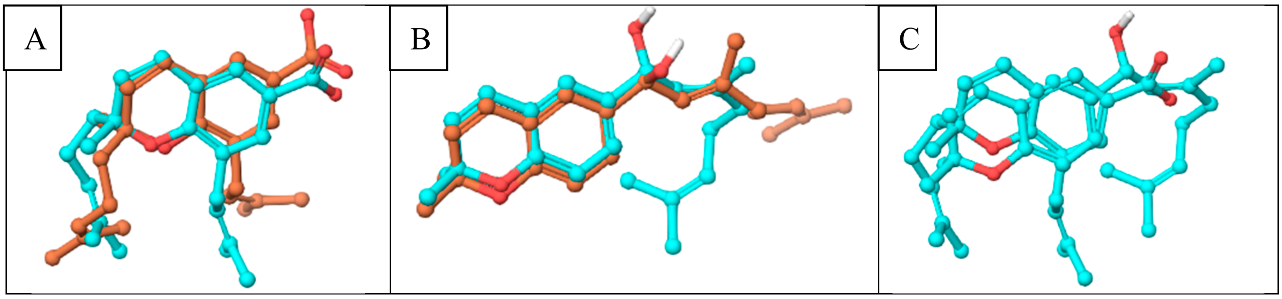

Figure S19. Superposition of docked conformations of *R* and *S* enantiomers, PL competitive inhibitors. A. ***3R***(cyan) and ***3S*** (brown); B. ***7R*** (cyan) and ***7S*** (brown); C.***3R*** and ***7R***.

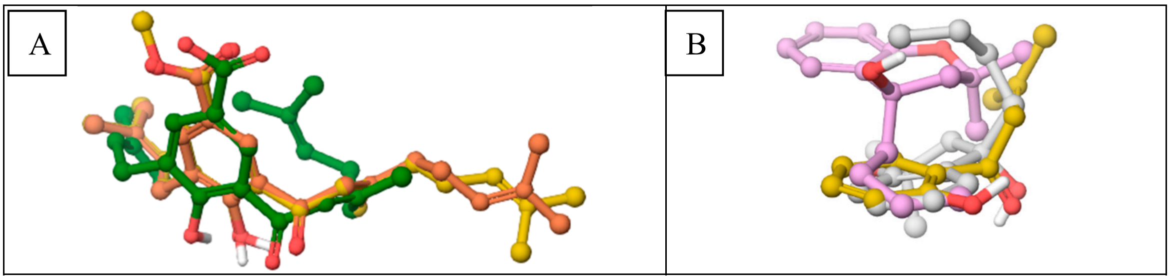

Figure S20. Superposition of docked conformations of PL inhibitors. A. **1** (orange), **2** (green) and **8** (yellow). B. **5** (Yellow), ***6R*** (pink), and ***6S*** (grey).

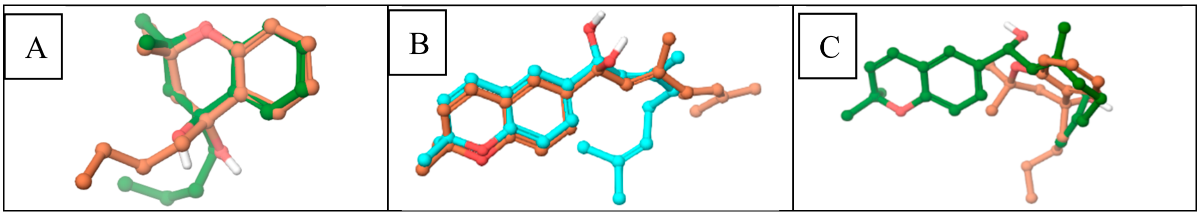

Figure S21. Superposition of docked conformations of AG inhibitors. A. ***6R*** (cyan) and ***6S*** (brown). B. ***7R*** (Yellow) ***7S***. C ***6R*** (orange), and ***7R*** (green).

Table S1. Binding free energies predicted by molecular docking between pancreatic lipase, α-glucosidase, and its compounds.

Compound	Pancreatic Lipase (LP)			Alpha-Glucosidase (AG)		
	Catalytic site	Allosteric site	Inhibition type	Catalytic site	Allosteric site	Inhibition type
	Binding energy (Kcal/mol)			Binding energy (Kcal/mol)		
**1**	--	-7,3	UC	--	--	--
**2**	--	-6,2	UC	--	--	--
**3R**	-8,7	--	C	--	--	--
**3S**	-8,6	--	C	--	--	--
**5**	--	-6,0	NC	--	--	--
**6R**	--	-6,6	NC	--	-6,1	M
**6S**	--	-6,3	NC	--	-6,0	M
**7R**	-8,9	--	C	--	-7,8	NC
**7S**	-8,7	--	C	--	-7,8	NC
**8**	--	-7,3	M	--	--	--

Table S2. Type of interactions in the binding sites of pancreatic lipase and α-glucosidase.

Interaction

Pancreatic Lipase — Aminoacid interaction

Compound										
**Catalytic Site**										
3R	77 Phe	78 Ile	79 Asp	152 Ser	178 Ala	215 Phe	256 Arg	259 Ala	260 Ala	263 His
3S	77 Phe	78 Ile	79 Asp	152 Ser	178 Ala	215 Phe	256 Arg	259 Ala	260 Ala	263 His
7R	77 Phe	78 Ile	79 Asp	152 Ser	178 Ala	215 Phe	256 Arg	259 Ala	260 Ala	263 His
7S	77 Phe	78 Ile	79 Asp	152 Ser	178 Ala	215 Phe	256 Arg	259 Ala	260 Ala	263 His
**Allosteric Site**										
1	41 Leu	42 Lys	64 Glu	229 Asn	235 Pro	337 Arg	369 Tyr	386 Phe	387 Ash	389 Asp
2	41 Leu	42 Lys	64 Glu	229 Asn	235 Pro	337 Arg	369 Tyr	386 Phe	387 Ash	389 Asp
5	25 Ser	41 Leu	42 Lys	64 Glu	65 Arg	367 Lys	368 Gln			
6R	25 Ser	41 Leu	42 Lys	64 Glu	65 Arg	367 Lys	368 Gln	369 Tyr	370 Glu	403 Tyr
6S	25 Ser	26 Asn	41 Leu	42 Lys	64 Glu	65 Arg	356 Leu	367 Lys	368 Gln	
8	41 Leu	42 Lys	64 Glu	229 Asn	328 Asp	329 Thr	369 Tyr	386 Phe	387 Ash	389 Asp

α-Glucosidase — Aminoacid interaction

Compound									
**Allosteric site**									
6R	636 Tyr	636 Tyr	639 Thr	653 Arg	676 Pro	733 Tyr	732 Gly	765 Lys	766 Gly
6S	636 Tyr	639 Thr	640 Leu	653 Arg	676 Pro	733 Tyr	732 Gly	766 Gly	767 Glu
7R	271 Glu	269 Thr	639 Thr	640 Leu	649 Asp	653 Arg	733 Tyr	766 Gly	767 Glu
7S	271 Glu	269 Thr	639 Thr	640 Leu	649 Asp	653 Arg	733 Tyr	766 Gly	767 Glu

Key data

Interaction type

Pi-Pi	(yellow)	*polar*	(red)
Hydrophobics	(green)	H -bond	(blue)

**Pancreatic lipase**				**α-glucosidase**		
Catalytic site	**77 Phe**	**263 His**	**152 Ser**	Catalytic site	**327 Asp**	**443 Asp**
